# Aortic arch calcification: A simple but powerful marker of subclinical cardiovascular disease

**DOI:** 10.1016/j.lanwpc.2022.100500

**Published:** 2022-06-15

**Authors:** Saadia Qazi, Michael L. Chuang

**Affiliations:** Division of Non-Invasive Cardiovascular Imaging, Brigham and Women's Hospital (SQ); Cardiovascular Division, Beth Israel Deaconess Medical Center (MLC), Boston, MA 02215, USA

The burden of cardiovascular disease (CVD) worldwide remains high despite advances in diagnosis and management.^1^ Risk factor modification has become the cornerstone of CVD prevention. Because the burden of CVD remains high throughout the world, it is important to identify subclinical markers of CVD, in addition to traditional clinical risk factors, to aid in risk stratification and prognostication. Coronary artery calcification (CAC), as well as calcifications in extracoronary vascular beds, have provided great insights into the risk of developing clinical CVD.^2^ However, there remains a relative paucity of data in non-Western populations that may have a different burden of subclinical disease and for whom the presence of subclinical vascular calcifications may translate to a different prognostic profile.

Tian and colleagues^3^ investigated the risk of adverse events associated with aortic arch calcification (AAC) on chest x-ray imaging. The study population comprised 27,166 adults from Guangzhou, China who were over 50 years of age and free of clinical CVD. Those with any AAC visualized on chest x-ray, were older (65.5 vs 59.9 years) and had a higher burden of CVD risk factors including hypertension, LVH by ECG criteria, diabetes, larger waist circumference, as well as higher levels of inflammatory markers such as uric acid and white blood cell counts. Over a mean 14.3-year follow-up, on multivariable Cox proportional hazards models adjusted for CVD risk factors and socioeconomic factors, there was a significant association between AAC and adverse events including all-cause mortality (HR 1.24, 95% CI 1.17-1.31) and non-fatal and fatal CVD (HR 1·22, 95% CI 1.14-1.30). There were a total of 5,350 deaths and 4,012 CVD events. The authors then additionally considered LVH by ECG criteria, as a surrogate marker of arterial stiffness and afterload-related vascular damage. AAC-and-LVH was associated greater hazard of all-cause mortality (HR 1.72, 95% CI 1.37 – 2.15) and adverse CVD event (HR 1.80, 95% CI 1.40 – 2.32) compared to those who had neither marker of subclinical CVD. Additionally, a higher burden of AAC resulted in a greater risk of both all-cause mortality and CVD events.

A similar study by Irribaren et al,^2^ investigated the association of AAC on chest x-ray with adverse events in an American cohort, which included 60,393 women and 55,916 men who underwent chest x-ray between 1964-1973. Over a median follow-up of 28 years, there was an increased risk of hospitalization or death from coronary heart disease (CHD), ischemic stroke, hemorrhagic stroke, and peripheral vascular disease among both men (relative risk (RR) 1.27 (95%CI 1.11-1.45) and women (relative risk (RR) 1.22 (95%CI 1.07-1.38). This was a predominantly Caucasian population. The present study by Tian et al, extends similar findings to a Chinese population. Further, Tian et al additionally considered ECG-LVH in conjunction with AAC, accounting for two key markers of vascular health,^1^^,^^3^ which have been previously studied in specific populations with renal disease.^4^^,^^5^

In addition to x-ray studies, CT-based extracoronary calcifications have been studied in large epidemiologic cohorts;^6^^,^^7^ however, these have included measures of thoracic aortic calcifications, but not AAC specifically. Bos et al,^8^ demonstrated that among 2408 elderly participants from the Rotterdam Study (mean age, 69.6 years), there was a 2.72-fold greater hazard (95% CI 1.85–4.02) of cardiovascular mortality per 1-SD increase in AAC. However, while AAC may have significant prognostic value, traditional coronary artery calcium assessments often do not capture the aortic arch.^1^ On dedicated extended CT scans, Craiem et al^9^ found that the aortic arch and proximal descending aorta harbored a significant burden of calcifications. In this context, the study by Tian et al emphasizes the value of obtaining AAC as a meaningful marker of subclinical CVD. Chest x-rays do not allow detailed quantification of AAC burden as does CT imaging, but from chest plain films AAC can be characterized as absent/present, or even graded (e.g. no AAC, total length of AAC<10mm, total length ≥10mm, as used by Tian et al) with minimal reading overhead.

The present study by Tian et al provides insight into the CVD and mortality risk associated with AAC in a community-dwelling Chinese cohort, which is valuable since large studies related to AAC are relatively limited among East Asian populations.

## Contributors

SQ and MLC contributed to the preparation and writing of the manuscript.

## Declaration of interests

The authors declare no conflict of interests.
